# Response Surface Optimization of Oil Removal Using Synthesized Polypyrrole-Silica Polymer Composite

**DOI:** 10.3390/molecules25204628

**Published:** 2020-10-11

**Authors:** Oisaemi Uduagele Izevbekhai, Wilson Mugera Gitari, Nikita Tawanda Tavengwa, Wasiu Babatunde Ayinde, Rabelani Mudzielwana

**Affiliations:** 1 Environmental Remediation and Nano Sciences Research Group, School of Environmental Sciences, University of Venda, Private Bag X5050, Thohoyandou 0950, South Africa; twasiu33@gmail.com (W.B.A.); mudzrabe@gmail.com (R.M.); 2 Department of Chemistry, University of Venda, Private Bag X5050, Thohoyandou 0950, South Africa; Nikita.Tavengwa@univen.ac.za

**Keywords:** adsorbent, water remediation, synthetic oily wastewater, central composite design, adsorption

## Abstract

The severity of oil pollution, brought about by improper management, increases daily with an increase in the exploration and usage of oil, especially with an increase in industrialization. Conventional oil treatment methods are either expensive or time consuming, hence the need for new technologies. The aim of this research is to synthesize polypyrrole-modified silica for the treatment of oily wastewater. Pyrrole was copolymerized with silica in the presence of ferric chloride hexahydrate by adding 23 mL of 117.4 g/dm^3^ ferric chloride hexahydrate drop wise to a silica-pyrrole mixture (1:2.3). The mixture was stirred for 24 h, filtered and dried at 60 °C for 24 h. The composite was then characterized using FTIR and SEM/EDX. A central composite model was developed in design expert software to describe the efficiency of oil removal using the polypyrrole-modified silica under the influence of initial oil concentration, adsorbent dosage and contact time. The synthesized adsorbent had FTIR bands at 3000–3500 cm^−1^ (due to the N-H), 1034 cm^−1^ (attributed to the Si-O of silica), 1607 cm^−1^ and 1615 cm^−1^ (due to the stretching vibration of C=C of pyrrole ring). The adsorption capacity values predicted by the central composite model were in good agreement with the actual experimental values, indicating that the model can be used to optimize the removal of oil from oily wastewater in the presence of polypyrrole-modified silica. The adsorbent showed excellent oil uptake when compared with similar materials. The optimum conditions for oil removal were 7091 mg/L oil concentration, 0.004 g adsorbent dosage and contact time of 16 h. Under these conditions, the percentage of oil adsorption was 99.3% and adsorption capacity was 8451 mg/g. As a result of the low optimum dosage and the lack of agitation, the material was found to be applicable in the remediation of field wastewater.

## 1. Introduction

Generally, oils in wastewater are classified as either dissolved or dispersed. Dissolved oils are either aromatics, acids or phenols, while dispersed oils are either aromatics, acids or aliphatics.

These groups of compounds are stable to light and heat, and as a result, they do not biodegrade easily and thus have a high pollution potential [1]. They are released into the environment during mining and transportation, thereby earning a top spot on the environmental issues list [2,3,4,5,6]. Methods that have been previously used in the removal of organic pollutants from water and in the treatment of oily wastewater are coagulation, filtration with coagulation, precipitation, ozonation, adsorption, ion exchange, reverse osmosis, biological methods and advanced oxidation processes. These methods, except for adsorption, are expensive to run, cannot be used without proper training, and in the case of biological methods, take up too much time [7].

Adsorption has proven to be one of the best methods in the remediation of oily wastewater, especially when the right adsorbent is employed [8]. An ideal adsorbent should have high adsorption capacity, high lipophilicity and low hydrophobicity, should be recyclable and should be environmentally friendly [9].

It has been somewhat of a challenge to fabricate the ideal adsorbent for oil removal so far [10]. Several natural adsorbents have been used for oil sorption. These include rice husk ash [11], carbonized peat bagasse [12], saw dust [13], vegetable fibers [14] and sugarcane bagasse [15]. One drawback of using natural adsorbents is that they are in short supply in some regions. Chemically modified natural adsorbents have also been used with some degree of success. For example, acetylated corncobs have been used in the remediation of oily water with a sorption capacity of 2500 mg/g [16]. Oleic acid-modified sawdust has also been used and was found to have relatively high sorption capacity. Inorganic materials have been applied to adsorb oil and these materials include fly ash, activated carbon, zeolite and vermiculite [17,18,19,20,21,22]. One drawback of the use of these materials, however, is the low adsorption ability. Other materials such as carbon nanotubes, filter papers, metal meshes, superhydrophilic and underwater superoleophobic metal meshes and diesel exhaust emission soot-coated polyurethane foams have been applied in the remediation of oily waters but these too have limitations, such as high costs and complex fabrication [23,24,25]. Synthetic adsorbents, like polydimethylsiloxane-coated polyurethane (PU) sponge, perfluorooctyltriethoxysilane—polypyrrole sponge, and polypyrrole—palmitic acid polyurethane sponge have also been used, with adsorption capacities reportedly higher than those of inorganic and natural adsorbents [10].

Composites that have been used in oil sorption include polyurethane hybrid composite [26], nanoporous carbon-silica composite [27] and natural rubber/reduced-graphene oxide composite materials [28], with adsorption capacities of 12.2 g/g, 0.4 g/g and 21 g/g, respectively. These materials have better oil sorption ability than non-composite materials because they become strongly hydrophobic and more lipophilic, leading to better oil sorption.

The silica precursor, diatomaceous earth, has been used in the raw and calcined form to adsorb different groups of oil such as benzene, toluene, ethylbenzenes, p-xylenes and o-xylenes, with adsorption capacities of 0.05–0.8 mg/g [29].

One common denominator of the adsorbents that have been used in oil sorption studies is the fact that they have low adsorption capacities and that it takes a long time to remove oil. Given the relatively low adsorption capacities, and the problems associated with the use of some of these sorbents, it is important to find relatively cheap and reusable adsorbents. Also, most studies focus on the ability of adsorbents to take up oil but do not look at the quality of water after adsorption in terms of the amount of oil left in solution. This study reports, for the first time, the modification of silica extracted from the abundant diatomaceous earth with polypyrrole which introduces a π electron system suitable for adsorption and its application in the remediation of synthetic oily wastewater (SOWW).

## 2. Results

### 2.1. Characterization of Polypyrrole—Silica Composite

**Figure 1:**
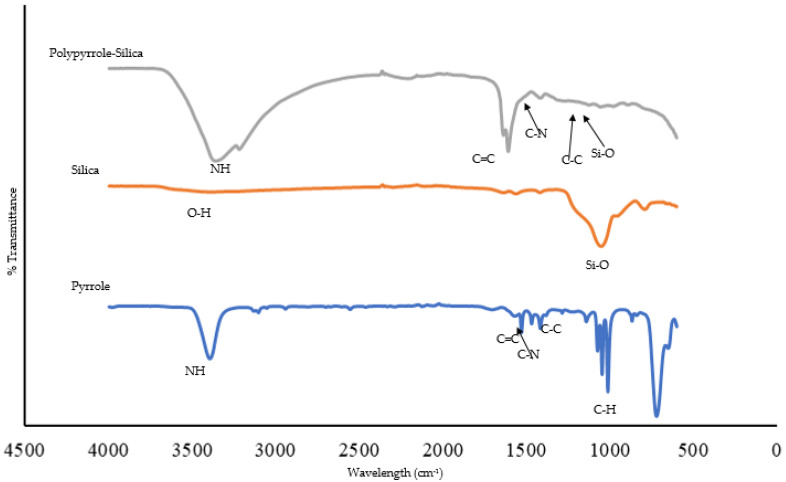
FTIR plots of pyrrole, silica and polypyrrole-silica composite.

The polypyrrole-silica composite was scanned in the range between 4000 and 500 cm^−1^ so as to ascertain the success of the composite preparation. The FTIR spectrum of the synthesized polypyrrole-silica composite (Figure 1) shows a broadband at about 3000–3500 cm^−1^ due to the N-H stretching vibration of pyrrole in the polymer. This band is broader than what is found in pyrrole, which shows a sharp band at 3390 cm^−1^. This is probably as a result of intermolecular hydrogen bonding. This is consistent with findings by Zhao et al. [30] and Yussuf et al. [31] who studied the synthesis of polypyrrole. The double peak at 1607 cm^−1^ and 1615 cm^−1^ could be due to the stretching vibration of the C=C of pyrrole ring. This band shifted from 1530 cm^−1^ in pyrrole to these positions in polypyrrole possibly as a result of increased conjugation. The transmission band due to C-N shifted from 1417 cm^−1^ in pyrrole to 1407 cm^−1^ in polypyrrole probably as a result of increased conjugation and intermolecular hydrogen bonding. The transmission band at about 1034 cm^−1^ (dwarfed by the other peaks) is attributed to the Si-O of silica which corresponds to the peak found in the spectra for silica.

**Figure 2:**
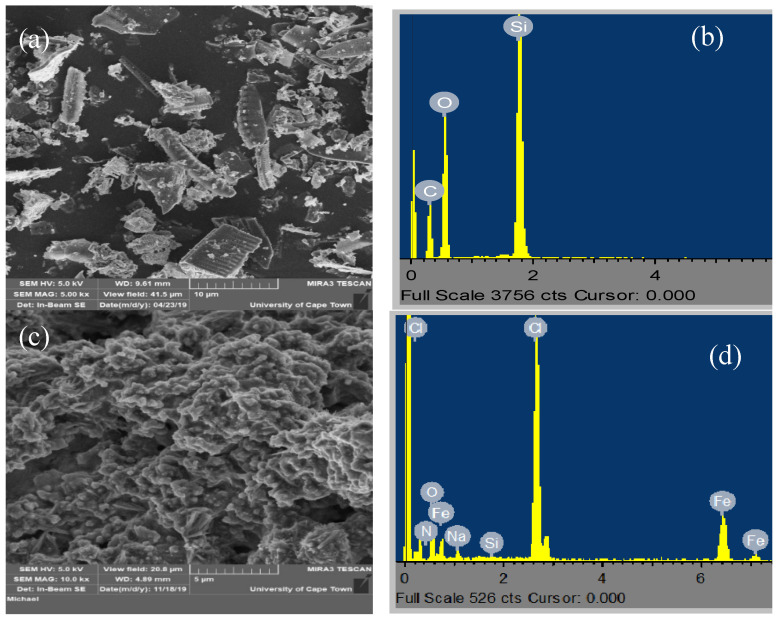
(**a**,**c**) SEM of unmodified and polypyrrole-silica composite, respectively, and (**b**,**d**) the corresponding EDX.

Figure 2a,c shows the SEM image of the unmodified silica and the as-prepared polypyrrole-Si complex, respectively. The unmodified silica (a) was seen to have razor-like crystals with well-defined external pores. This appearance changed to a one-dimensional morphology intertwined together in the polypyrrole-modified silica (c). The rough surface suggests that the material will be a good adsorbent. The EDX of the unmodified silica (b) showed silica and oxygen as the major elements, while the polypyrrole-silica composite (d) showed the presence of silica and elements such as nitrogen and oxygen which make up the pyrrole ring, indicating successful functionalization. This is consistent with findings by Zhao et al. [30].

### 2.2. Optimization of Oil Removal

#### 2.2.1. RSM Experiments and Model Fitting

Modelling of the percentage removal of oil from SOWW was done using the quadratic model of the central composite design of the response surface methodology (RSM) software (version 11, Stat-Ease Inc., Minneapolis, MN, USA) employing three reaction parameters, namely, initial oil concentration (mg/L), adsorbent dosage (g) and contact time (h). The actual percentage removal, as calculated using Equation (1.2), is presented in Table 1.

The color of the adsorbent-treated water had an inverse relationship with the percentage of oil adsorption. The more intense the color, the lower the percentage removal and vice versa. The trends observed in the table are as described in the response surface plots in Section 2.2.3.

**Figure 3:**
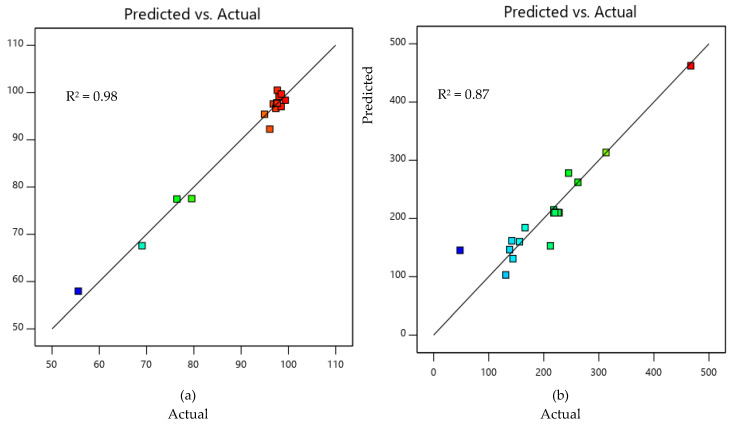
Comparison between values predicted by the RSM model and experimentally determined values for (**a**) percentage adsorption and (**b**) total organic carbon.

The model predictions for percentage removal of oil from SOWW and total organic carbon of the adsorbent-treated SOWW were compared with the values obtained from experiments and plotted in Figure 3a,b to obtain a coefficient of determination R^2^ of 0.98 and 0.87 for percentage removal and total organic carbon, respectively. This indicated that predicted and actual values were in good agreement and that the quadratic polynomial model chosen was sufficient to explain the relationship between the responses and significant variables [32,33,34]. The red color on the graphs represents points that were above the predicted values, the green-colored points were equal to the predicted values, while the light and dark-blue points were below the predicted values.

#### 2.2.2. Regression Model and Analysis of Variance (ANOVA)

In order to test the significance of the model and the model terms, ANOVA was conducted (Table 2). It was observed that the quadratic polynomial model used to represent both total organic carbon (TOC) and percentage adsorption were significant (at *p*-value less than 0.05 and high F-value). Model terms like initial oil concentration, adsorbent dosage, interaction between adsorbent dosage and contact time and the quadratic function of oil concentration and contact time had an effect on the total organic carbon, while changes in initial oil concentration, adsorbent dosage, contact time and the interaction between these and their quadratic function had an effect on the percentage of oil removed.

#### 2.2.3. Effect of Oil Concentration and Sorbent Dosage

**Figure 4:**
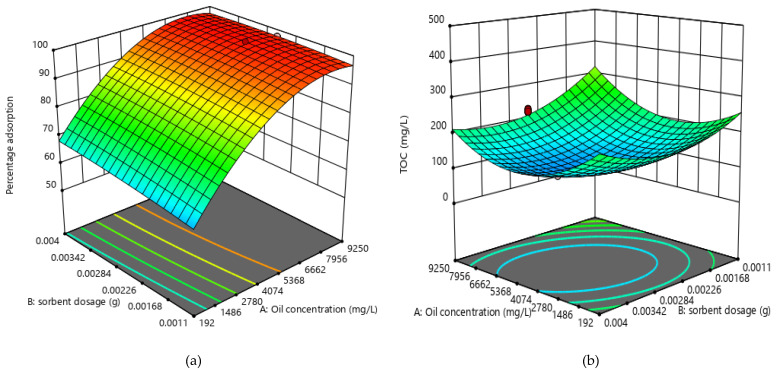
Response surface plots for effect of oil concentration and adsorbent dose for (**a**) percentage adsorption (**b**) TOC.

Figure 4 shows the response surface plot for oil concentration and sorbent dosage and their interaction on the TOC of the adsorbent-treated SOWW and the percentage removal of oil by the polypyrrole-silica composite. As observed, the percentage of oil removal increased steadily up to 99%, which was the maximum with an increase in initial oil concentration, and then decreased to 55% with a further increase in initial oil concentration. When initial oil concentration was increased, the oil droplets increased and passed into the pores of the adsorbent easily, hence the initial increase in percentage adsorption [35,36,37]. Furthermore, as adsorbent dosage was increased, percentage adsorption increased slightly. This was likely because of the introduction of new binding sites on the adsorbents. Generally, as initial oil concentration increases, the net ability of adsorbents to remove oil decreases because the binding sites on the adsorbents eventually become saturated and the total organic carbon decreases [38]. Hassan et al. [38] and Rahdar et al. [39] found similar trends when they applied polyacrylamide in the treatment of refinery wastewater and modified saxaul ash in the adsorption of arsenic (v), respectively.

#### 2.2.4. Effect of Oil Concentration and Time

**Figure 5:**
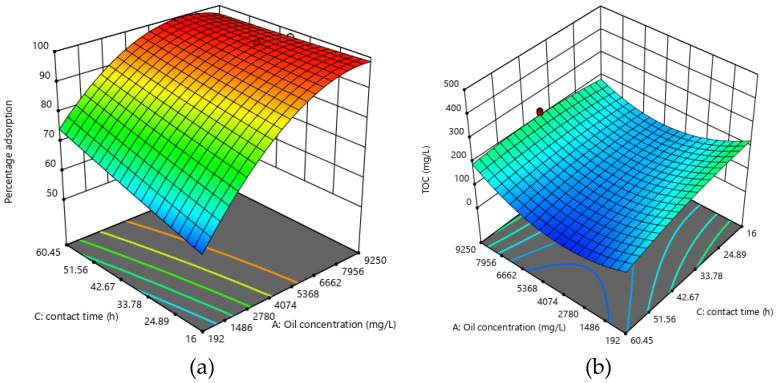
Response surface plots for effect of oil concentration and time for (**a**) percentage adsorption (**b**) TOC.

As can be seen from the response surface plots in Figure 5, percentage of oil removal increased with time. This is because there is an increased chance of positive interaction between the adsorbent and oil as the adsorbent stayed longer in contact with the solution. Izevbekhai et al. [40] also found the same trend when they applied acetylated silica in the treatment of SOWW. The opposite is true of TOC as reported by Hassan et al. [38]. 

#### 2.2.5. Effect of Adsorbent Dosage and Time

**Figure 6:**
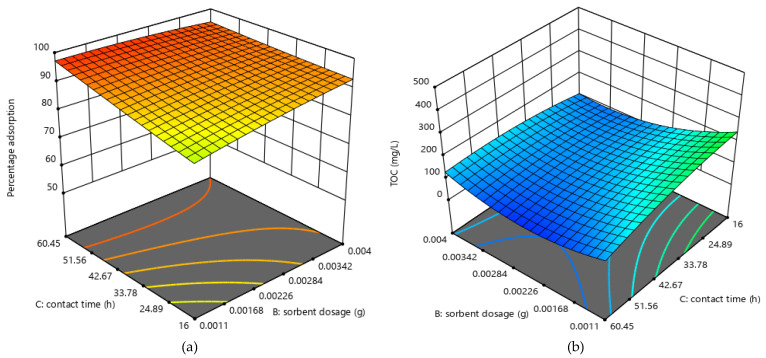
Response surface plots for effect of contact time and adsorbent dosage for (**a**) percentage adsorption (**b**) TOC.

Figure 6 illustrates the response surface plot for adsorbent dose and time, and the interaction on TOC and percentage oil removal. Percentage adsorption was found to increase with both time and adsorbent dosage. The opposite was true of TOC, but with adsorbent dosage, a minimum was reached, and percentage adsorption increased. As the dosage was increased, more binding sites were made available, and as time was increased, there was more time for the oil molecules to interact with the binding sites. Therefore, at higher doses, more oil is adsorbed, since there are more empty binding sites leading to lower TOC values [39].

## 3. Comparison to Other Adsorbents

Table 3 compares the adsorption capacity of the polypyrrole-silica complex synthesized in this study with other reported adsorbents. It is noted that the adsorption capacity of the synthesized polypyrrole adsorbent is higher than most similar adsorbents but lower than the polypyrrole-coated cigarette filters. However, it is common knowledge that the adsorption capacity is dependent on the type and concentration of adsorbent used. Zhang et al. [41] used 2 mol/L pyrrole, while 0.004 mol/L was used in this study. Also, all the literature studies reported here and almost all studies available in the literature were only concerned about how much oil the adsorbents were able to take up [16,19,30,36,41,42,43], but this study looked mostly at the quality of the adsorbent-treated water. Considering this, polypyrrole-silica composite is a promising adsorbent for oily wastewater treatment.

## 4. Blank Effect

**Figure 7:**
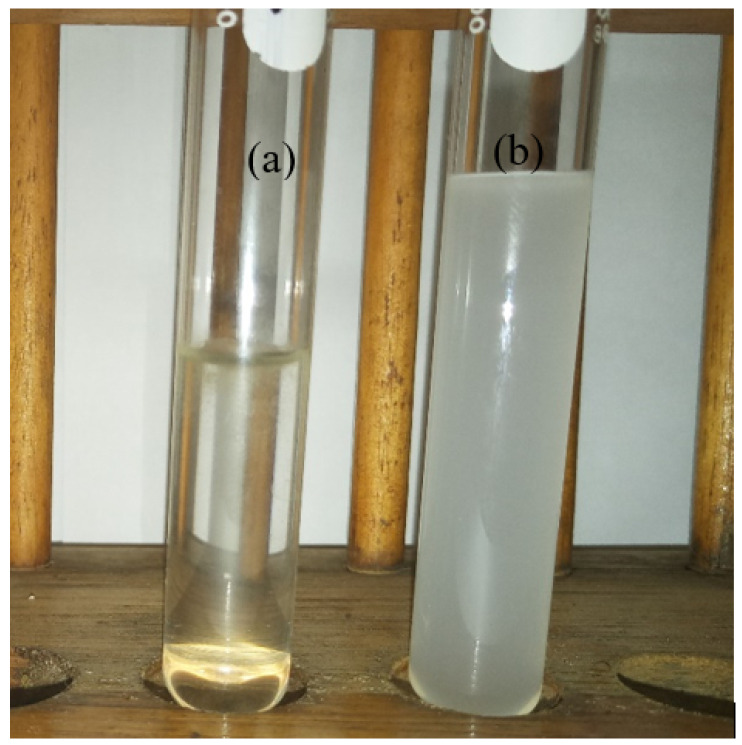
(**a**) Adsorbent-treated SOWW (**b**) empty tea bag-treated SOWW (initial oil concentration—6650 mg/L; duration–16 h.

The effect of empty tea bags was studied by placing sealed tea bags of similar shape and size in SOWW of the same concentrations as used in Table 1. It was observed visually, as seen in Figure 7, that oil removal with sealed tea bags alone was not very effective and that the contribution of the sealed tea bags alone was negligible. The adsorbent-treated SOWW in Figure 7a is clear as a result of the action of the adsorbents on the oil in the water, while the Figure 7b is ‘milky’ because the functional groups necessary for effective oil sorption are absent on the empty tea bags.

## 5. Materials and Methods

### 5.1. Materials

Silica aerogel previously extracted from diatomaceous earth was used after drying to constant weight [44], pyrrole, sodium dodecylsulphate, iron (III) chloride were of analytical grade and obtained from Sigma-Aldrich (St Louis, MO, USA). Vacuum pump oil was obtained from Telstar Technologies (Barcelona, Spain). Milli-Q water from Millipore S.A.S (Molsheim, France) (18.2 µS/cm at 25 °C) was also used in all dilutions. Tea bags were obtained from retail outlets in Thohoyandou, South Africa. A Telstar Lyoquest-55 freeze dryer (Shangai, China) was used to freeze dry all samples and a Spectroquant UV spectrophotometer from Merck Group (Germiston, South Africa) was used for total organic carbon testing and a Stuart reciprocating shaker (Staffordshire, UK) was used for shaking. An ultrasonic processor (UP 4005) from Hielscher ultrasound technology, with amplitude of 20 to 100% and cycle of 0.1–1 (Berlin, Germany) was used in wastewater preparation, and an ALPHA Fourier-transform infrared spectrophotometer from Bruker Pty (Sandton, South Africa) was used in functional group analysis.

### 5.2. Preparation of Polypyrrole-Modified Silica

The polypyrrole-modified silica was prepared by modifying the method used by Zhao et al. [30]. Typically, two solutions were prepared. Solution A was prepared by adding 0.3 g silica to 0.7 mL pyrrole, and solution B was prepared by adding 2.7 g of ferric chloride hexahydrate to 23 mL of deionized water while stirring. Solution B was added dropwise to solution A while stirring at room temperature. Stirring was continued for 24 h, after which the solution was filtered, the solute was washed with deionized water and dried in an oven at 60 °C for 12 h.

### 5.3. Characterization of Synthesized Silica Composite

FTIR with transmission mode was used to investigate the chemical composition of pyrrole, silica and polypyrrole functionalized silica. The measurements were carried out using an ALPHA FT-IR spectrophotometer and were recorded in the range of 400–600 cm^−1^. The morphology of the composite (size and shape at the surface) was investigated by sprinkling a small amount of the sample onto an SEM stub covered with carbon glue. The stubs were then coated with carbon in an evaporation coater. The SEM is a NanoSEM 230 (FEI Nova, Brno, Czechoslovakia Republic) with a field emission gun (FEG). The elemental analysis was carried out using an X-Max detector (Oxford, Abingdon, UK) equipped with Inca software (version 7.2, ETAS, Derby, UK), at 20 kV.

### 5.4. Preparation of Synthetic Oily Wastewater

Synthetic oily wastewater (SOWW) was prepared using the method described by Shoba, Jeyanthi and Vairam [43]. Vacuum pump oil was mixed with sodium dodecyl sulphate (90:1, *v*/*w*) in 1 liter of water, sonicated for 5 min at an amplitude of 75% and cycle of 0.5 to obtain a homogenous solution. This was used in response surface optimization experiments.

### 5.5. Response Surface Optimization of Oil Removal

**Figure 8:**
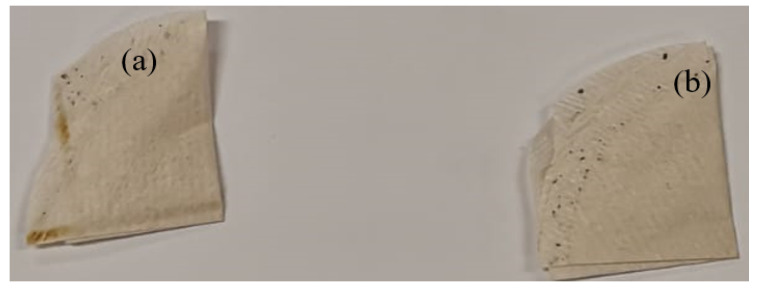
(**a**) Sealed empty tea bag, (**b**) sealed tea bag containing adsorbents.

The procedure for optimization was carried out using the central composite design of the design expert software (version 11, Stat-Ease Inc., Minneapolis, USA) by placing a variable amount of the prepared adsorbent in an empty tea bag whose content had been emptied out prior to the experiments (Figure 8b). To account for the effect of the tea bags and sealing of the tea bags, empty tea bags were also sealed and used in similar experiments (Figure 8a). The tea bags were then placed in the SOWW (prepared in Section 5.4) and allowed to stand ‘undisturbed’ for some time. After this, the solution was filtered, and the total organic carbon was measured for the initial SOWW and adsorbent-treated SOWW using a Spectroquant UV spectrophotometer. The equilibrium adsorption was calculated using Equation (1) and percentage removal using Equation (2).

(1) $$Q_{e}=\left(\frac{\left(C_{i}-C_{f}\right)}{m}\right)V.$$

(2) $$\%\;\mathrm{Removal}=\left(\frac{\left(C_{i}-C_{f}\right)}{C_{i}}\right)100$$

where C_i_ and C_f_ are the oil concentrations (mg/L) before and after treatment with adsorbent, respectively; V (L) is the volume of the solution, m (g) is the mass of the adsorbent and Q_e_ is the equilibrium adsorption capacity per milligram dry weight of the adsorbent.

### 5.6. Experimental Design

Experiments for the optimization of oil removal were done by varying the weight of the adsorbent, oil concentration and contact time and were then correlated with responses obtained from Section 5.5 by the central composite design in the RSM software (version 11, Stat-Ease Inc., Minneapolis, MN, USA). The range of optimized parameters are listed in Table 4. The range of oil concentration was chosen based on the concentration of oil in actual oil spills.

## 6. Conclusions

Polypyrrole-modified silica has been designed by the copolymerization of pyrrole and silica in the presence of ferric chloride hexahydrate and employed in the remediation of synthetic oily wastewater using an adsorbent-in-tea-bag method. The response surface model, the central composite design which was developed to describe the efficiency of oil removal, revealed that initial oil concentration, adsorbent dosage and contact time, their interaction and the quadratic function of initial oil concentration determined the uptake of oil with initial oil concentration having the most impact on oil adsorption. The optimum conditions for the removal of oil from SOWW were 7091 mg/L oil concentration, 0.004 g adsorbent dosage and contact time of 16 h with a desirability of 0.95. The percentage of oil adsorption was found to be 99.3% and the adsorption capacity under these conditions was found to be 8451 mg/g. Experiments were completed without any form of agitation, leaving the treated water clear, and the adsorption capacity of the adsorbent in the removal of oil from SOWW was comparable to other adsorbents reported in the literature. Given this, the synthesized adsorbent would be applicable in field wastewater. It is, however, recommended that the eco-friendliness of the adsorbent be tested before application in the field.

## Figures and Tables

**Table 1:** Response surface methodology (RSM) design and the actual values of responses.

Run	Initial Oil Concentration (mg/L)	Sorbent Dosage (g)	Contact Time (h)	Final Oil Concentration (mg/L)	Experimental Percentage Adsorption
1	6650	0.004	16	131	98
2	9250	0.003	38.2	226	97.6
3	704	0.001	60.5	144	79.5
4	9250	0.003	0.85	138	98.5
5	704	0.004	60.5	166	76.4
6	9250	0.003	38.2	220	97.6
7	704	0.004	16	218	69
8	6650	0.001	60.5	156	97.6
9	192	0.003	38.2	194	−1.04
10	9250	0.003	38.2	228	97.5
11	704	0.001	16	313	55.5
12	9250	0.005	38.2	245	97.3
13	7250	0.003	38.2	48	99.3
14	6650	0.001	16	262	96
15	6650	0.004	60.5	212	96.8
16	9250	0.0001	38.2	467	95
17	9250	0.003	38.2	222	97.6
18	9250	0.003	38.2	218	97.6
19	9250	0.003	75.6	142	98.5
20	9250	0.003	38.2	220	97.6

**Table 2:** ANOVA for applied model and model terms.

	Total Organic Carbon (TOC)		% Removal	
Source	F-Value	*p*-Value	F-Value	*p*-Value
Model	6.9	0.0041	63.15	<0.0001
A-Oil concentration	0.3955	0.545	457.32	<0.0001
B-sorbent dosage	6.53	0.0309	5.29	0.047
C-contact time	4.4	0.0653	32	0.0003
AB	2.4	0.1558	2.04	0.1874
AC	4.23	0.0698	33.54	0.0003
BC	6.17	0.0347	10	0.0115
A^2^	8.07	0.0194	49.96	<0.0001
B^2^	22.38	0.0011	1.01	0.341
C^2^	2.72	0.1335	0.1358	0.721

**Table 3:** Comparison of adsorption capacity for different adsorbents. SOWW: synthetic oily wastewater.

Adsorbent	Type of Oil Studied	Sorption Capacity (mg/g)	Reference
Acetylated corncobs	Oily water	2500	Nwadiogbu et al. [16]
Silica aerogels	Oil adsorption	3500	Filipovic et al. [42]
Polypyrrole-coated cigarette filters	Engine oil	13,600	Zhang et al. [41]
Polypyrrole-Si complex	SOWW	8451	This study

**Table 4:** Range of optimized parameters.

Parameter	Minimum	Maximum
Oil concentration (mg/L)	192	9250
Sorbent dosage (g)	0.0011	0.004
Contact time (h)	16	60.45
